# Supplementary material for: The association of plasma osteoprotegerin levels and functional outcomes post endovascular thrombectomy in acute ischemic stroke patients: a retrospective observational study
Source: PeerJ. 2022 May 3;10:e13327. doi: 10.7717/peerj.13327 (PMC9074858; doi:10.7717/peerj.13327)
Supplement: Supplemental Information 1 [file peerj-10-13327-s001.docx]

**Supplementary Table 1.** Correlation of tertile for the plasma OPG concentrations with other variables

| **Variables** | **Osteoprotegerin levels, pg/mL** | | | |
| --- | --- | --- | --- | --- |
|  | **Tertile 1 (n=122)**  **29.89 ~ 166.04** | **Tertile 2 (n=113)**  **166.04 ~ 239.25** | **Tertile 3 (n=125)**  **239.25 ~ 490.26** | ***p* value** |
| Demographics |  |  |  |  |
| Sex, male | 73 (59.8) | 45 (39.8) | 67 (53.6) | 0.008 |
| Age, years | 74.5 ± 13.6 | 75.5 ± 13.9 | 74.9 ± 14.2 | 0.649 |
| Body mass index, kg/m^2^ | 21.1 ± 3.8 | 20.3 ± 4.2 | 20.2 ± 4.0 | 0.067 |
| Risk factors |  |  |  |  |
| Hypertension | 89 (73.0) | 87 (77.0) | 93 (74.4) | 0.772 |
| Diabetes mellitus | 64 (52.5) | 58 (51.3) | 67 (53.6) | 0.940 |
| Hypercholesterolaemia | 52 (42.6) | 60 (53.1) | 52 (41.6) | 0.149 |
| Coronary artery disease | 32 (26.2) | 33 (29.1) | 32 (25.6) | 0.803 |
| Congestive heart failure | 9 (7.4) | 9 (8.0) | 9 (7.2) | 0.973 |
| Atrial fibrillation | 59 (48.4) | 57 (50.4) | 66 (52.8) | 0.784 |
| Smoking | 19 (15.6) | 10 (8.8) | 29 (23.2) | 0.011 |
| Alcohol intake | 33 (27.0) | 29 (25.7) | 25 (20.0) | 0.392 |
| Previous stroke history | 30 (24.6) | 44 (38.9) | 30 (24.0) | 0.017 |
| Prior medication |  |  |  |  |
| Anti-platelet | 37 (30.3) | 40 (35.4) | 42 (33.6) | 0.702 |
| Anti-coagulant | 24 (19.4) | 30 (26.5) | 18 (14.4) | 0.064 |
| Statins | 39 (32.0) | 42 (37.2) | 36 (28.8) | 0.383 |
| NIHSS | 13 [9 – 18] | 14 [11 – 19] | 15 [11 – 20] | 0.011 |
| Thrombectomy procedure |  |  |  |  |
| Thrombolysis methods |  |  |  | 0.670 |
| Mechanical thrombectomy only | 69 (56.6) | 70 (61.9) | 76 (60.8) |  |
| tPA and mechanical thrombectomy | 53 (43.4) | 43 (38.1) | 49 (39.2) |  |
| Onset-to-puncture time (min) | 370.3 ± 352.6 | 301.6 ± 252.4 | 344.5 ± 285.5 | 0.321 |
| Number of trials for thrombectomy | 2 [1 – 3] | 2 [1 – 3] | 2 [1 – 3] | 0.594 |
| Recannalization (TICI IIb or III) | 107 (87.7) | 97 (85.8) | 107 (85.6) | 0.872 |
| Hemorrhagic transformation |  |  |  | 0.111 |
| No hemorrhagic transformation | 84 (68.9) | 76 (67.3) | 70 (56.0) |  |
| HI1 | 16 (13.1) | 17 (15.0) | 12 (9.6) |  |
| HI2 | 10 (8.2) | 9 (8.0) | 18 (14.4) |  |
| PH1 | 5 (4.1) | 5 (4.4) | 12 (9.6) |  |
| PH2 | 7 (5.7) | 6 (5.3) | 13 (10.4) |  |
| Any hemorrhagic transformation | 38 (31.1) | 37 (32.7) | 55 (44.0) | 0.073 |
| Stroke subtype |  |  |  | 0.073 |
| Cardioembolism | 54 (44.3) | 66 (58.4) | 70 (56.0) |  |
| Large artery atherosclerosis | 20 (16.4) | 21 (18.6) | 17 (13.6) |  |
| Undetermined two or more causes | 25 (20.5) | 8 (7.1) | 15 (12.0) |  |
| Undetermined negative | 18 (14.8) | 10 (8.8) | 16 (12.8) |  |
| Other determined | 5 (4.1) | 8 (7.1) | 7 (5.6) |  |
| Blood laboratory findings |  |  |  |  |
| Vitamin D 25(OH)D, ng/mL | 21.1 ± 6.5 | 20.2 ± 7.0 | 20.8 ± 7.2 | 0.637 |
| Glucose at admission, mg/dL | 146.6 ± 54.0 | 135.8 ± 36.5 | 139.0 ± 45.3 | 0.958 |
| Triglyceride, mg/dL | 113.9 ± 75.2 | 109.3 ± 66.8 | 111.4 ± 55.7 | 0.475 |
| Total cholesterol, mg/dL | 164.4 ± 44.7 | 159.4 ± 47.3 | 166.1 ± 39.5 | 0.242 |
| Low-density lipoprotein, mg/dL | 94.6 ± 36.0 | 91.4 ± 37.7 | 99.0 ± 35.3 | 0.152 |
| White blood cell count, ×10^3^ | 8.7 ± 5.5 | 8.1 ± 3.2 | 8.0 ± 2.7 | 0.736 |
| Hemoglobin, mg/dL | 13.7 ± 1.9 | 12.8 ± 2.1 | 13.3 ± 2.0 | 0.003 |
| Creatinine, mg/dL | 0.9 ± 0.7 | 0.8 ± 0.5 | 0.8 ± 0.3 | 0.300 |
| Total calcium, mg/dL | 8.2 ± 0.4 | 8.2 ± 0.4 | 8.2 ± 0.3 | 0.604 |
| Phosphate, mg/dL | 3.2 ± 0.5 | 3.1 ± 0.6 | 3.0 ± 0.5 | 0.231 |
| C-reactive protein, mg/L | 0.6 ± 1.1 | 0.8 ± 1.4 | 1.0 ± 3.4 | 0.765 |

Data are shown as n (%), mean ± standard deviation or median [interquartile range].

mRS: modified Rankin Scale, NIHSS: National Institute of Health Stroke Scale, tPA: tissue plasminogen activator, TICI: Thrombolysis in cerebral infarction, HI: hemorrhagic infarction, PH: parenchymal hematoma.
