# Supplementary material for: The association of plasma osteoprotegerin levels and functional outcomes post endovascular thrombectomy in acute ischemic stroke patients: a retrospective observational study
Source: PeerJ. 2022 May 3;10:e13327. doi: 10.7717/peerj.13327 (PMC9074858; doi:10.7717/peerj.13327)
Supplement: Supplemental Information 2 [file peerj-10-13327-s002.docx]

**Supplementary Table 2.** Multivariate binary logistic regression analysis for the association of osteoprotegerin levels with functional outcomes

| Variables | Univariate binary  logistic regression  OR (95% CI) | Multivariate binary logistic regression  OR (95% CI) | | |
| --- | --- | --- | --- | --- |
|  |  | Model 1 | Model 2 | Model 3 |
| Demographics and risk factors |  |  |  |  |
| Sex, male | 0.890 (0.584 – 1.357) | 1.818 (0.964 – 3.488)^†^ | 1.818 (0.964 - 3.488)^†^ | 1.950 (1.022 - 3.796)^*^ |
| Age, years | 1.031 (1.014 – 1.049)^*^ | 1.025 (1.002 - 1.050)^*^ | 1.025 (1.002 - 1.050)^*^ | 1.026 (1.003 - 1.050)^*^ |
| Body mass index, kg/m^2^ | 0.957 (0.907 – 1.009) | 0.970 (0.895 - 1.049) | 0.970 (0.895 - 1.049) | 0.966 (0.892 - 1.046) |
| Diabetes mellitus | 2.989 (1.930 – 4.681)^*^ | 2.000 (1.108 - 3.647)^*^ | 2.000 (1.108 - 3.647)^*^ | 2.034 (1.124 - 3.718)^*^ |
| NIHSS | 1.170 (1.123 – 1.224)^*^ | 1.183 (1.123 - 1.253)^*^ | 1.183 (1.123 - 1.253)^*^ | 1.176 (1.116 - 1.246)^*^ |
| Thrombolysis related factors |  |  |  |  |
| Thrombolysis methods |  |  |  |  |
| Mechanical thrombectomy only | Reference | Reference | Reference | Reference |
| tPA and mechanical thrombectomy | 0.573 (0.367 – 0.885)^*^ | 0.683 (0.383 - 1.210) | 0.683 (0.383 - 1.210) | 0.690 (0.386 - 1.226) |
| Number of trials for thrombectomy | 1.179 (1.044 – 1.338)^*^ | 1.001 (0.857 - 1.176) | 1.001 (0.857 - 1.176) | 1.009 (0.862 - 1.187) |
| Recanalization (TICI IIb or III) | 0.173 (0.083 – 0.335)^*^ | 0.100 (0.038 - 0.241)^*^ | 0.100 (0.038 - 0.241)^*^ | 0.104 (0.040 - 0.248) ^*^ |
| Any hemorrhagic transformation | 2.179 (1.406 – 3.392)^*^ | 1.976 (1.122 - 3.505)^*^ | 1.976 (1.12 – 3.50)^*^ | 1.925 (1.091 - 3.419)^*^ |
| Blood laboratory findings |  |  |  |  |
| White blood cell count | 1.047 (0.993 – 1.112) | 1.051 (0.977 – 1.128) | 1.051 (0.977 – 1.128) | 1.055 (0.980 - 1.133) |
| Vitamin D 25(OH)D | 0.955 (0.925 – 0.985)^*^ | 0.966 (0.927 - 1.005)^†^ | 0.966 (0.927 - 1.005)^†^ | 0.967 (0.928 - 1.006) |
| Glucose at admission | 1.005 (1.001 – 1.010)^*^ | 1.004 (0.998 – 1.010) | 1.004 (0.998 – 1.010) | 1.004 (0.998 - 1.010) |
| Total cholesterol | 0.996 (0.991 – 1.000)^†^ | 0.998 (0.990 – 1.005) | 0.998 (0.990 – 1.005) | 0.998 (0.991 - 1.005) |
| Hemoglobin | 0.858 (0.772 – 0.952)^*^ | 0.858 (0.735 – 0.996)^*^ | 0.858 (0.735 – 0.996)^†^ | 0.858 (0.735 – 0.997)^†^ |
| C-reactive protein | 1.207 (1.034 – 1.468)^*^ | 1.346 (1.089 – 1.755)^*^ | 1.346 (1.089 – 1.755)^*^ | 1.336 (1.085 - 1.750)^*^ |

Data are shown as OR (95% CI). ^*^*p*<0.05, ^†^*p*<0.1

OR: odds ratio, CI: confidence interval, NIHSS: National Institute of Health Stroke Scale, tPA: tissue plasminogen activator, TICI: thrombolysis in cerebral infarction, SD: standard deviation.

^a^Adjusted for sex, body mass index, and variables with p values <0.1 in the univariate analysis (age, NIHSS, DM, thrombolysis methods, number of trials for thrombectomy, successful recanalization, any hemorrhagic transformation, blood glucose level at admission, hemoglobin, total cholesterol, WBC, C-reactive protein, and vitamin D 25(OH)D)

Models 1, 2, 3 are adjusted for OPG levels as continuous variables, per standard deviation and categorical variable (tertiles), respectively
