# Supplementary material for: The association of plasma osteoprotegerin levels and functional outcomes post endovascular thrombectomy in acute ischemic stroke patients: a retrospective observational study
Source: PeerJ. 2022 May 3;10:e13327. doi: 10.7717/peerj.13327 (PMC9074858; doi:10.7717/peerj.13327)
Supplement: Supplemental Information 3 [file peerj-10-13327-s003.docx]

**Supplementary Table 3.** Multivariate ordinal logistic regression analysis for the association of osteoprotegerin levels with functional outcomes

| Variables | Univariate ordinal  logistic analysis  OR (95% CI) | Multivariate ordinal logistic regression  OR (95% CI) | | |
| --- | --- | --- | --- | --- |
|  |  | Model 1 | Model 2 | Model 3 |
| Demographics and risk factors |  |  |  |  |
| Sex, male | 0.711 (0.493 - 1.026)^†^ | 1.029 (0.656 - 1.615) | 1.029 (0.656 - 1.615) | 1.135 (0.720 - 1.790) |
| Age, years | 1.026 (1.012 - 1.039)^*^ | 1.019 (1.004 - 1.035)^*^ | 1.019 (1.004 - 1.035)^*^ | 1.020 (1.005 - 1.036) ^*^ |
| Body mass index, kg/m^2^ | 0.946 (0.904 - 0.990)^*^ | 0.985 (0.931 - 1.042) | 0.985 (0.931 - 1.043) | 0.981 (0.927 - 1.038) |
| Diabetes mellitus | 2.913 (2.000 - 4.264)^*^ | 1.842 (1.192 - 2.851)^*^ | 1.842 (1.193 - 2.851)^*^ | 1.883 (1.220 - 2.915)^*^ |
| NIHSS | 1.156 (1.119 - 1.194)^*^ | 1.150 (1.111 - 1.193)^*^ | 1.151 (1.111 - 1.193)^*^ | 1.145 (1.105 - 1.187)^*^ |
| Thrombolysis related factors |  |  |  |  |
| Thrombolysis methods |  |  |  |  |
| Mechanical thrombectomy only |  |  |  |  |
| tPA and mechanical thrombectomy | 0.534 (0.365 - 0.777)^*^ | 0.584 (0.387 - 0.878)^*^ | 0.584 (0.387 - 0.878)^*^ | 0.585 (0.388 - 0.880)^*^ |
| Number of trials for thrombectomy | 1.181 (1.065 - 1.311)^*^ | 1.027 (0.918 - 1.148) | 1.027 (0.919 - 1.148) | 1.034 (0.923 - 1.158) |
| Recanalization (TICI IIb or III) | 0.184 (0.104 - 0.324)^*^ | 0.153 (0.082 - 0.280)^*^ | 0.153 (0.082 - 0.280)^*^ | 0.153 (0.082 - 0.279)^*^ |
| Any hemorrhagic transformation | 2.295 (1.565 - 3.379)^*^ | 1.818 (1.203 - 2.754)^*^ | 1.818 (1.203 - 2.754)^*^ | 1.807 (1.192 - 2.745)^*^ |
| Blood laboratory findings |  |  |  |  |
| White blood cell count | 1.033 (0.990 - 1.084) | 1.027 (0.982 - 1.076) | 1.028 (0.982 - 1.076) | 1.033 (0.987 - 1.082) |
| Vitamin D 25(OH)D | 0.955 (0.930 - 0.981)^*^ | 0.963 (0.936 - 0.991)^*^ | 0.963 (0.936 - 0.991)^*^ | 0.965 (0.937 - 0.993)^*^ |
| Glucose at admission | 1.005 (1.001 - 1.009) ^†^ | 1.004 (0.999 - 1.008)^†^ | 1.004 (0.999 - 1.008)^†^ | 1.004 (0.999 - 1.008) ^†^ |
| Total cholesterol | 0.997 (0.992 - 1.000) | 1.000 (0.996 - 1.005) | 1.000 (0.996 - 1.005) | 1.001 (0.996 - 1.005) |
| Hemoglobin | 0.867 (0.793 - 0.948)^*^ | 0.901 (0.811 - 0.999)^†^ | 0.901 (0.811 - 0.999)^†^ | 0.900 (0.810 - 0.998)^†^ |
| C-reactive protein | 1.094 (1.012 - 1.190)^*^ | 1.147 (1.055 - 1.250)^*^ | 1.147 (1.055 - 1.250)^*^ | 1.150 (1.057 - 1.252)^*^ |

Data are shown as OR (95% CI). ^*^*p*<0.05, ^†^*p*<0.1

OR: odds ratio, CI: confidence interval, NIHSS: National Institute of Health Stroke Scale, tPA: tissue plasminogen activator, TICI: thrombolysis in cerebral infarction, SD: standard deviation.

^a^Adjusted for sex, body mass index, and variables with p values <0.1 in the univariate analysis (age, NIHSS, DM, thrombolysis methods, number of trials for thrombectomy, successful recanalization, any hemorrhagic transformation, blood glucose level at admission, hemoglobin, total cholesterol, WBC, C-reactive protein, and vitamin D 25(OH)D)

Models 1, 2, 3 are adjusted for OPG levels as continuous variables, per standard deviation and categorical variable (tertiles), respectively
