# Supplementary material for: The association of plasma osteoprotegerin levels and functional outcomes post endovascular thrombectomy in acute ischemic stroke patients: a retrospective observational study
Source: PeerJ. 2022 May 3;10:e13327. doi: 10.7717/peerj.13327 (PMC9074858; doi:10.7717/peerj.13327)
Supplement: Supplemental Information 4 [file peerj-10-13327-s004.docx]

**Supplementary Table 4** Comparison of model fitness

|  | AUC | *p* value | NRI | *p* value | IDA | *p* value |
| --- | --- | --- | --- | --- | --- | --- |
| Variables^a^  without OPG level | 0.855 | Reference | Reference |  | Reference |  |
| Variables^a^  with OPG level | 0.859 | 0.379 | 0.012 | 0.027 | 0.174 | 0.104 |

^a^Variables are sex, body mass index, and variables with p values <0.1 in the univariate analysis (age, NIHSS, DM, thrombolysis methods, number of trials for thrombectomy, successful recanalization, any hemorrhagic transformation, blood glucose level at admission, hemoglobin, total cholesterol, WBC, C-reactive protein, vitamin D 25(OH)D)

AUC: area under the curve, IDI: Integrated Discrimination Index, NRI: Net Reclassification Improvement
