# Supplementary material for: The association of plasma osteoprotegerin levels and functional outcomes post endovascular thrombectomy in acute ischemic stroke patients: a retrospective observational study
Source: PeerJ. 2022 May 3;10:e13327. doi: 10.7717/peerj.13327 (PMC9074858; doi:10.7717/peerj.13327)
Supplement: Supplemental Information 5 [file peerj-10-13327-s005.docx]

**Supplementary Table 5.** Multivariable binary logistic analysis for association of osteoprotegerin levels with functional outcome

| **Variables** | **Door to puncture time**  **< 480 minute** | **Door to puncture time**  **≥ 480 minute** |
| --- | --- | --- |
| Demographics and risk factors |  |  |
| Sex, male | 0.903 (0.557 – 1.461) | 0.817 (0.336 – 1.977) |
| Age, years | 1.033 (1.014 – 1.054)^*^ | 1.023 (0.990 – 1.060) |
| Body mass index, kg/m^2^ | 0.941 (0.884 – 1.000) | 1.006 (0.904 – 1.121) |
| Diabetes mellitus | 2.917 (1.766 – 4.893)^*^ | 3.538 (1.434 – 9.113)^*^ |
| NIHSS | 1.208 (1.146 – 1.280)^*^ | 1.113 (1.034 – 1.207)^*^ |
| Thrombolysis related factors |  |  |
| Thrombolysis methods |  |  |
| Mechanical thrombectomy only | Reference | Reference |
| tPA and mechanical thrombectomy | 0.576 (0.353 – 0.935)^*^ | 0.400 (0.019 – 3.281) |
| Number of trials for thrombectomy | 1.183 (1.035 – 1.360)^*^ | 1.177 (0.882 – 1.644) |
| Recannalization (TICI IIb or III) | 0.200 (0.088 – 0.421)^*^ | 0.106 (0.016 – 0.434)^*^ |
| Any hemorrhagic transformation | 2.052 (1.247 – 3.390)^*^ | 2.766 (1.091 – 7.281)^*^ |
| Blood laboratory findings |  |  |
| Osteoprotegerin per 1 SD | 1.551 (1.199 – 2.030)^*^ | 0.863 (0.541 – 1.355) |
| Vitamin D 25(OH)D | 0.958 (0.924 – 0.992)^*^ | 0.942 (0.878 – 1.007)^†^ |
| Glucose at admission | 1.004 (1.000 – 1.009)^†^ | 1.016 (1.003 – 1.034)^*^ |
| Total cholesterol | 0.995 (0.989 – 1.001)^†^ | 0.998 (0.987 – 1.007) |
| Hemoglobin | 0.851 (0.754 – 0.957)^*^ | 0.866 (0.679 – 1.088) |
| C-reactive protein | 1.232 (1.039 – 1.535)^*^ | 1.124 (0.709 – 1.937) |

Data are shown as OR (95% CI). ^*^*p*<0.05, ^†^*p*<0.1

OR: odds ratio, CI: confidence interval, NIHSS: National Institute of Health Stroke Scale, tPA: tissue plasminogen activator, TICI: Thrombolysis in cerebral infarction, SD: standard deviation.
