# Supplementary material for: The association of plasma osteoprotegerin levels and functional outcomes post endovascular thrombectomy in acute ischemic stroke patients: a retrospective observational study
Source: PeerJ. 2022 May 3;10:e13327. doi: 10.7717/peerj.13327 (PMC9074858; doi:10.7717/peerj.13327)
Supplement: Supplemental Information 6 [file peerj-10-13327-s006.docx]

**Supplementary Methods**

***Clinical variables***

A detailed definition of risk factors for hypertension was described in a previous study (Song et al. 2017). In brief, hypertension was defined as being present when a patient had been taking blood pressure-lowering agents, or had a resting systolic blood pressure ≥140 mmHg or diastolic blood pressure ≥90 mmHg on repeated measurements. Diabetes mellitus was diagnosed when the patient had a fasting blood glucose level ≥7.0 mmol/L, or was being treated with oral glucose-lowering medications or insulin. Hypercholesterolaemia was diagnosed if the patient had total cholesterol ≥6.2 mmol/L, low-density lipoprotein cholesterol ≥4.1 mmol/L, or if the patient had taken lipid-lowering medications after a diagnosis of hyperlipidemia. Coronary artery disease was defined as a history of myocardial infarction, unstable angina, or angiographically confirmed coronary artery occlusive disease. Patients were defined as smokers if they were current smokers or had stopped smoking within 1 year before the index stroke. Subjects whose recent mean weekly alcohol intake had regularly exceeded 300 g of ethanol were classified as heavy drinkers (Song et al. 2016).

**References**

Song TJ, Kim YD, Yoo J, Kim J, Chang HJ, Hong GR, Shim CY, Song D, Heo JH, and Nam HS. 2016. Association between Aortic Atheroma and Cerebral Small Vessel Disease in Patients with Ischemic Stroke. *J Stroke* 18:312-320. 10.5853/jos.2016.00171

Song TJ, Park JH, Choi KH, Kim JH, Choi Y, Chang Y, Kim HJ, Moon J, Kim YJ, and Lee HW. 2017. Is obstructive sleep apnea associated with the presence of intracranial cerebral atherosclerosis? *Sleep Breath* 21:639-646. 10.1007/s11325-016-1450-9
